# Content Differences of Active Ingredients in Different Flower Colors of *Carthamus tinctorius* L. and Their α-Glucosidase Inhibitory Effects Based on Network Pharmacology and Molecular Docking

**DOI:** 10.3390/cimb48070746

**Published:** 2026-07-22

**Authors:** Fan Huang, Wenzheng Zhao, Shiqing Wang, Jun Li, Ling Zeng, Jingfang Zhu

**Affiliations:** 1 College of Food Science and Pharmacy, Xinjiang Agricultural University, Urumqi 830052, China; hffan0822@126.com (F.H.);; 2 Xinjiang Yumin County Agricultural Technology Extension Station, Tacheng 834800, China

**Keywords:** *Carthamus tinctorius* L., different flower colors, active ingredients, α-glucosidase, network pharmacology, molecular docking

## Abstract

This study investigated the content differences of major active ingredients in red, yellow, and white flowers of *Carthamus tinctorius* L. from Yumin County, Xinjiang, and evaluated their α-glucosidase inhibitory effects using high-performance liquid chromatography (HPLC), network pharmacology, molecular docking, and in vitro enzyme assays. HPLC analysis revealed that red safflower had the highest content of Hydroxysafflor yellow A (HSYA), while white safflower exhibited the highest levels of kaempferol and kaempferol-3-O-rutinoside. Network pharmacology identified 21 core targets and multiple pathways related to type 2 diabetes and insulin resistance. Molecular docking indicated that kaempferol-3-O-rutinoside had the lowest binding energy (−9.3 kcal/mol). However, in vitro assays showed that kaempferol exhibited the strongest α-glucosidase inhibition (half maximal inhibitory concentration (IC_50_) = 0.1768 mg/mL), followed by HSYA and kaempferol-3-O-rutinoside. Among extracts, white safflower demonstrated the highest inhibitory activity (IC_50_ = 0.5485 mg/mL). In conclusion, flower color significantly influences the chemical composition and hypoglycemic potential of safflower. White safflower extract exhibited the strongest α-glucosidase inhibitory activity among the three flower color extracts. This study identifies kaempferol as the key active ingredient for safflower’s α-glucosidase inhibitory activity, providing a scientific basis for developing Yumin safflower as hypoglycemic functional food.

## 1. Introduction

Diabetes mellitus, a highly prevalent metabolic disease worldwide, is witnessing a continuously and alarmingly rising incidence, among which type 2 diabetes accounts for more than 90% of all diabetes cases [1]. Controlling postprandial blood glucose levels is a key aspect of diabetes management, and α-glucosidase, as the critical enzyme in the small intestine that catalyzes the breakdown of carbohydrates into glucose, has become an important therapeutic target [2]. Acarbose, currently used in clinical practice, exhibits significant inhibitory effects on α-glucosidase; however, its administration is commonly associated with gastrointestinal adverse reactions such as abdominal distension and diarrhea [3]. Therefore, the search for novel α-glucosidase inhibitors with high efficacy and low toxicity from natural products has become a research hotspot in recent years.

Safflower refers to the dried flower of *Carthamus tinctorius* L., a plant of the Asteraceae family, also known as “thorn safflower” or “grass safflower” [4]. According to the Compendium of Materia Medica (Bencao Gangmu), safflower exhibits the effects of promoting blood circulation, unblocking meridians, removing blood stasis, and alleviating pain [5]. China is one of the major producing countries of safflower. As a multi-purpose economic crop used for food, medicine, dye, and feed, safflower possesses high practical value and broad prospects for development [6]. Modern studies have shown that safflower exerts various pharmacological effects, including anticoagulation, antithrombosis, antitumor activity, increasing coronary blood flow, vasodilation, improving microcirculation, and regulating immunity [7]. Compounds such as flavonoids, alkaloids, organic acids, and steroids have been isolated and identified from safflower. Safflower is rich in flavonoids and flavonoid glycosides, among which the main active ingredients closely related to its pharmacological effects are HSYA and kaempferol [8]. In the Chinese Pharmacopoeia (2020 edition, Volume I), HSYA and kaempferol are used as marker components for content determination [9]. In addition, the kaempferol derivative kaempferol-3-O-rutinoside can also be detected in safflower and is present at relatively high levels [10]. HSYA possesses anti-ischemic, antioxidant, neuroprotective, and anti-inflammatory activities [11]. Kaempferol exhibits multiple effects, including antioxidant, anti-inflammatory, antitumor, anti-osteoporotic, anti-cerebral ischemia, and hypoglycemic properties [12]. Kaempferol-3-O-rutinoside shows antioxidant, anti-inflammatory, hypoglycemic, and neuroprotective activities [13]. To systematically elucidate the hypoglycemic mechanisms of these active components, network pharmacology and molecular docking were employed in this study.

Yumin County, located in Tacheng Prefecture in the northwestern part of the Xinjiang Uygur Autonomous Region, is renowned as “the hometown of China’s thornless safflower” [14]. Safflower serves as a pillar industry in this region, with a planting area exceeding 100,000 mu (approximately 6667 hectares). The unique geographical environment, along with favorable conditions of water, soil, light, and heat, provides excellent support for safflower cultivation and endows the local thornless safflower with its distinctive quality [15]. Although Yumin County yields a high output of safflower, there have been few studies focusing on safflower with different flower colors in this area.

However, a critical knowledge gap remains: although the bioactive components of safflower are well documented, no study has systematically compared the contents of Hydroxysafflor yellow A (HSYA), kaempferol, and kaempferol-3-O-rutinoside among red, yellow, and white flower phenotypes from Yumin County, nor has the consequent variation in α-glucosidase inhibitory activity been evaluated. Given that flower color in safflower is known to be associated with differential flux in flavonoid biosynthesis, we hypothesize that the three color variants possess distinct chemical profiles, and that white safflower, predicted to accumulate higher levels of kaempferol, will exhibit the strongest α-glucosidase inhibitory effect. Testing this hypothesis will not only clarify the relationship between flower color and bioactivity but also provide a scientific rationale for selecting specific color types for functional food development.

Therefore, high-performance liquid chromatography (HPLC) was used to determine the contents of three components (HSYA, kaempferol, and kaempferol-3-O-rutinoside) in safflower samples with three different flower colors. Combined with network pharmacology, the inhibitory effects of these samples on α-glucosidase were compared, and molecular docking technology was employed for validation in this study. This study provides a basis for evaluating the efficacy of safflower with different flower colors and its application in the prevention and treatment of diabetes mellitus, aiming to offer a foundation for the further development of safflower products in Yumin County.

## 2. Materials and Methods

### 2.1. Reagents and Materials

Three stable safflower cultivars with distinct flower colors, namely Yuhong No. 1 (red), Xinhonghua No. 4 (yellow), and Xinhonghua No. 7 (white), were used in this study. These cultivars were independently developed, officially registered, and cultivated in Yumin County, Tacheng, Xinjiang, China. Floral materials were harvested in July 2023. For each cultivar, a large quantity of flowers was randomly collected from numerous individual plants across the cultivation field to ensure representativeness. The collected samples were thoroughly mixed, homogenized, and then subsampled for subsequent analyses. The species was identified as *Carthamus tinctorius* L. by Prof. Jingfang Zhu from Xinjiang Agricultural University and Senior horticulturist Shiqing Wang from the Agricultural Extension Station of Yumin County, Xinjiang, based on morphological characteristics.

HSYA reference standard (purity above 98%, C15502993) was from Shanghai Macklin technology Co., Ltd. (Shanghai, China). Kaempferol (purity above 98.00%, A01HB190000) was obtained from Shanghai Yuanye Bio-Technology Co., Ltd. (Shanghai, China). Methanol (Sigma-Aldrich, St. Louis, MO, USA), acetonitrile (Sigma-Aldrich, St. Louis, MO, USA) and phosphoric acid (Sinopharm Group Co., Ltd., Taicang, China) were chromatographic grade. Methanol (Tianjin Xinbote Chemical Co., Ltd., Tianjing, China) was analytically reagent. Phosphate buffer solution powder was obtained from Solarbio Biotechnology Co., Ltd.(Beijing, China). α-Glucosidase, 4-Nitrophenyl-α-D-glucopyranoside (PNPG), and acarbose were obtained from Shanghai Yuanye Bio-Technology Co., Ltd. (Shanghai, China). Sodium carbonate (Na_2_CO_3_, analytical grade) was obtained from Tianjin Xinbote Chemical Co., Ltd. (Tianjin, China). Hydrochloric acid (HCl, analytical grade, 36–38%) and phosphoric acid (H_3_PO_4_, analytical grade) were obtained from Sinopharm Chemical Reagent Co., Ltd. Purified water was provided by Wahaha Group Co., Ltd. (Hangzhou, China).

### 2.2. Determination of the Content of Active Components in Different Safflowers

The HPLC experiment was performed using an Essentia SHIMADZU LC-16 (Kyoto, Japan), which was fitted using a binary pump (LC-16), manual injector (P/N 7725i-049) with a loop, column oven (CTO-16L), diode array detector (SPD-M20A), and LabSoiutions software (version 5.93). All samples were analyzed on a GL Sciences InertSustain C18 column (250 mm × 4.6 mm, 5 μm). The contents of HSYA and kaempferol in the samples were determined according to the method of the Chinese Pharmacopoeia (2020 Edition) [9] with slight modifications. The specific chromatographic conditions for HSYA were as follows: the mobile phase consisted of methanol–acetonitrile–0.5% phosphoric acid solution (26:2:72, *v*/*v*/*v*); the flow rate was set at 1.0 mL/min; the detection wavelength was 403 nm; and the column temperature was maintained at 35 °C. The specific chromatographic conditions for kaempferol were as follows: the mobile phase consisted of methanol–0.4% phosphoric acid solution (52:48, *v*/*v*); the flow rate was set at 1.0 mL/min; the detection wavelength was 367 nm; and the column temperature was maintained at 35 °C. The contents of kaempferol-3-O-rutinoside in the samples was determined by reference to YU’s method [16]. The chromatographic conditions for the determination of kaempferol-3-O-rutinoside were as follows: the mobile phase consisted of acetonitrile–0.2% phosphoric acid solution (15:85, *v*/*v*); the flow rate was set at 1.0 mL/min; the detection wavelength was 265 nm; and the column temperature was maintained at 35 °C.

#### 2.2.1. Preparation of Reference Standard Solutions

According to the specifications of the 2020 edition of the Chinese Pharmacopoeia [9], reference standard solutions of HSYA and kaempferol were prepared separately as follows: HSYA was dissolved in 25% methanol to obtain a solution with a concentration of 0.13 mg/mL; kaempferol was dissolved in pure methanol to obtain a solution with a concentration of 9 μg/mL. The reference standard solution of kaempferol-3-O-rutinoside was prepared by dissolving the compound in pure methanol to yield a final concentration of 50.4 μg/mL.

#### 2.2.2. Preparation of the Test Solution

The test sample solutions of HSYA and kaempferol were prepared in accordance with the methods stipulated in the 2020 edition of the Chinese Pharmacopoeia [9], while the test sample solution of kaempferol-3-O-rutinoside was prepared according to the method described by YU [16].

Preparation of the HSYA test sample solution: Approximately 0.4 g of safflower powder (passed through a No. 3 sieve) was accurately weighed and placed in a stoppered conical flask. A 50 mL aliquot of 25% methanol solution was added precisely, and the mixture was weighed before undergoing ultrasonic extraction at a power of 300 W and a frequency of 50 kHz for 40 min. After cooling to room temperature, the flask was weighed again, and the lost weight was replenished with 25% methanol. The mixture was then shaken well, filtered, and the subsequent filtrate was collected as the test sample solution.

Preparation of the kaempferol test sample solution: Approximately 0.5 g of safflower powder (passed through a No. 3 sieve) was accurately weighed, and 25 mL of methanol was added. After weighing, the mixture was heated under reflux for 30 min. Following cooling and weighing, the lost weight was replenished with methanol, and the mixture was shaken well and filtered. A 15 mL aliquot of the subsequent filtrate was precisely transferred to a flat-bottomed flask, followed by the addition of 5 mL of hydrochloric acid solution (prepared by diluting 15 mL of concentrated hydrochloric acid to 37 mL). The solution was mixed thoroughly and hydrolyzed in a water bath at 95 °C for 30 min. After rapid cooling, the hydrolysate was transferred to a 25 mL volumetric flask, diluted to volume with methanol, shaken well, and filtered. The resulting filtrate was collected as the test sample solution.

Preparation of the kaempferol-3-O-rutinoside test sample solution: Approximately 0.2 g of safflower powder (passed through a No. 3 sieve) was accurately weighed, and 25 mL of methanol was added. The mixture was sealed and subjected to ultrasonication for 40 min. After cooling, the lost weight was replenished with methanol, and the mixture was filtered. An appropriate volume of the extract was precisely drawn, diluted to 10 mL with methanol, and centrifuged. The supernatant was collected as the test sample solution.

#### 2.2.3. Establishment of the Standard Curve

An appropriate amount of the HSYA reference standard was accurately weighed and dissolved in 25% methanol to prepare a stock solution, which was subsequently diluted with the same solvent to yield a series of reference standard solutions at different concentrations. Appropriate amounts of the kaempferol and kaempferol-3-O-rutinoside reference standards were individually weighed accurately and dissolved in methanol to prepare respective stock solutions, which were then diluted with methanol to obtain a series of reference standard solutions of kaempferol and kaempferol-3-O-rutinoside at various concentrations. The reference standard solutions of the three constituents at different concentrations were analyzed individually under their respective chromatographic conditions. The corresponding chromatographic peak areas were determined, and the regression equations for the three constituents were established separately. The concentration ranges for each standard curve are summarized in Table 1. A single series of standard solutions was prepared for each calibration curve. Detailed concentration points and calibration data are provided in the Supplementary Materials (Supplementary Tables S1–S3).

#### 2.2.4. Sample Content Determination

The test sample solutions of safflower were prepared according to the aforementioned methods and analyzed under their respective chromatographic conditions to determine the contents of the three active constituents in each safflower sample. All determinations were performed in triplicate for each test sample, and the results are expressed as Mean ± SD.

### 2.3. Prediction of the Inhibitory Effects and Mechanisms of the Main Active Components of Safflower on α-Glucosidase Based on Network Pharmacology

#### 2.3.1. Acquisition of Three Major Active Ingredient Targets in Safflower

The canonical SMILES of the three main active components (HSYA, kaempferol, and kaempferol-3-O-rutinoside) were input into the SwissTargetPrediction database for target prediction. The prediction option was set to Homo sapiens. The obtained targets were merged and deduplicated to generate a set of component-related targets (*p* > 0.1) [17].

#### 2.3.2. Collection of α-Glucosidase-Related Targets

Using the GeneCards database, the keyword “Alpha-glucosidase” was used to search for targets (Relevance score > 10) associated with α-glucosidase [18].

#### 2.3.3. Screening of Main Active Components of Safflower and α-Glucosidase Related Targets

The relevant targets of the main active components and those associated with α-glucosidase were input into the Venny 2.1 web tool to obtain the mapping of intersecting targets between the main active components and α-glucosidase [19].

#### 2.3.4. The Interaction Network of Active Ingredient-Related Target Proteins Was Constructed

The protein–protein interaction (PPI) network of the intersecting targets between the active components and α-glucosidase was constructed using the STRING platform. The organism was set to Homo sapiens, and the intersecting targets were input as queries. The minimum required interaction score was set to the “medium confidence” threshold of 0.4, while other parameters were kept as default. The resulting network data were exported in TSV format and then imported into Cytoscape software (version 3.9.1) for visualization [20].

#### 2.3.5. GO Function and KEGG Pathway Enrichment Analysis

Using the DAVID database, Gene Ontology (GO) functional enrichment analysis and Kyoto Encyclopedia of Genes and Genomes (KEGG) pathway enrichment analysis were conducted on the protein-coding genes corresponding to the intersecting targets between the principal active constituents and α-glucosidase. A significance threshold of *p* < 0.05 and false discovery rate (FDR) < 0.05 was applied as the screening criterion for significant enrichment, with the aim of elucidating the roles of the relevant targets in terms of gene function and signaling pathways. The enrichment analysis results were subsequently visualized using the Weishengxin online platform [21].

#### 2.3.6. The Active Ingredient–Key Targets–Important Pathway Network Diagram Was Constructed

Data and attribute files for active components, key targets, and important pathways related to antioxidant and hypoglycemic activities were established separately. These files were then imported into Cytoscape software (version 3.9.1) to construct and visualize the “active component–key target–important pathway” network [22].

### 2.4. Molecular Docking Verification of the Active Ingredient and α-Glucosidase

The three-dimensional (3D) structures of the main active components of safflower and acarbose were obtained from the PubChem database. The 3D crystal structure of α-glucosidase(PDB ID 5NN8) was retrieved from the RCSB Protein Data Bank (PDB). Molecular docking between the main active components of safflower and α-glucosidase was performed using AutoDock Tools 1.5.7 software, and the docking results were visualized using PyMol software 3.1.0 [23].

### 2.5. In Vitro Inhibition of α-Glucosidase by Safflower Extracts and Their Main Active Components

The preparation procedure for the safflower extract was as follows: safflower was mixed with water at a solid-to-liquid ratio of 1:30 (g/mL), followed by extraction in a water bath maintained at 65 °C with ultrasonication at a power of 300 W for a duration of 2 h. The resulting extract solution was subjected to vacuum filtration, concentrated to a thick extract by rotary evaporation at 70 °C, and subsequently dried under reduced pressure at 50 °C to obtain the extract powder.

The in vitro inhibitory rates of acarbose, red safflower extract, yellow safflower extract, white safflower extract, HSYA, kaempferol, and kaempferol-3-O-rutinoside against α-glucosidase were determined individually using an enzyme–substrate reaction assay. In a 96-well microplate, 50 μL of 0.1 mol/L phosphate buffer solution (pH 7.4) was added, followed by 50 μL of α-glucosidase solution (0.5 U/mL). Then, 50 μL of the test sample was added. The mixture was incubated at 37 °C for 10 min. Subsequently, 50 μL of 10 mmol/L p-nitrophenyl-α-D-glucopyranoside (pNPG) solution was added, and the reaction was further incubated at 37 °C for 15 min. Finally, 50 μL of 0.2 mol/L Na_2_CO_3_ solution was added to terminate the reaction. The absorbance (A) was measured at 405 nm using a microplate reader. Acarbose was used as a positive control. The control (blank for inhibition) consisted of the reaction mixture without the inhibitor, and the blank control consisted of the reaction mixture without both the inhibitor and α-glucosidase. Three parallel experiments were performed. The inhibition rate was calculated according to Formula (1) [24].

(1) $$\mathrm{IR}\left(\%\right)=\left[1-\frac{A_{S}-A_{\mathrm{BS}}}{A_{C}-A_{\mathrm{BC}}}\right]\times 100\%$$

AS: absorbance of sample group; ABS: absorbance of sample blank group; AC: absorbance of control group; ABC: absorbance of blank control group.

### 2.6. Data Processing and Plotting

All content determination assays and in vitro enzyme experiments were performed in triplicate. Data are expressed as the mean ± standard deviation (Mean ± SD).

The half-maximal inhibitory concentration (IC_50_) values were calculated by fitting the inhibition rate curves using GraphPad Prism 9.0 (GraphPad Software, San Diego, CA, USA) with a non-linear regression model (log(inhibitor) vs. normalized response—variable slope). The corresponding inhibition curves were plotted using the same software.

Statistical analysis: Statistical comparisons among the three flower color groups (red, yellow, and white safflower) and among the three active components (HSYA, kaempferol, and kaempferol-3-O-rutinoside) were performed using one-way analysis of variance (ANOVA) followed by Tukey‘s honest significant difference (HSD) post hoc test for multiple comparisons, using SPSS 27.0 (IBM Corp., Armonk, NY, USA). A *p*-value < 0.05 was considered statistically significant. In the tables, different superscript letters (a, b, c) within the same column indicate significant differences (*p* < 0.05), while the same letters indicate no significant difference (*p* ≥ 0.05). All figures were generated using GraphPad Prism 9.0.

## 3. Results

### 3.1. Content Determination Results of the Main Active Components in Safflower with Three Different Flower Colors

#### 3.1.1. HPLC Chromatograms of the Three Reference Standards and Safflower Samples

**Figure 1:**
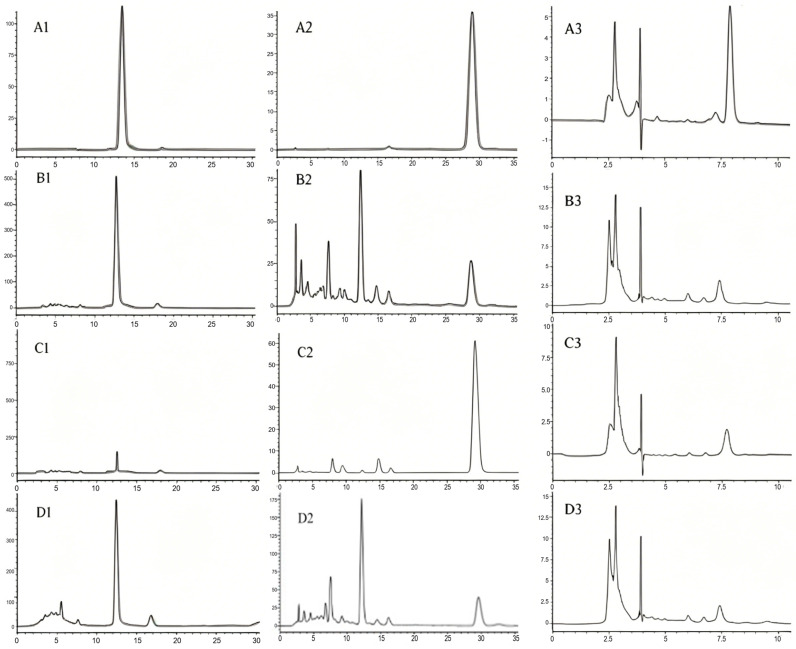
HPLC chromatograms of three active components in the three color varieties of safflowers. Note: 1, 2 and 3 represent HSYA, kaempferol, and kaempferol-3-O-rutinoside, respectively; (**A**–**D**) represent reference standards, red safflower, yellow safflower, and white safflower, respectively.

In Figure 1, panels A1–A3 represent the reference standards of HSYA, kaempferol, and kaempferol-3-O-rutinoside, respectively; panels B1–B3 show the chromatograms of the three components in red safflower; panels C1–C3 show those in white safflower; and panels D1–D3 show those in yellow safflower. As can be seen from the figure, the three components exhibited fast elution times and good peak shapes, with resolutions > 1.5 and theoretical plate numbers > 5000. The retention times of the peaks in the safflower samples were consistent with those of the corresponding reference standards. Furthermore, the chromatograms of HSYA and kaempferol complied with the requirements of the Chinese Pharmacopoeia [9].

#### 3.1.2. Establishment of Standard Curves

The linear regression equations of the three reference standards are shown in Table 1. HSYA, kaempferol, and kaempferol-3-O-rutinoside exhibited good linear relationships between peak area and concentration within the ranges of 4.95–296.7, 6.12–71.40, and 6.55–131.04 μg/mL, respectively, with correlation coefficients (r) all greater than 0.9995.

#### 3.1.3. Content Determination Results of the Three Active Components in Safflower

According to the Chinese Pharmacopoeia (2020 edition), the content of HSYA in red safflower shall not be less than 1.0%, and the content of kaempferol shall not be less than 0.050% [9]. The determination results of HSYA, kaempferol, and kaempferol-3-O-rutinoside in safflower of different colors from different origins are shown in Table 2. Only red safflower met both pharmacopoeial standards for HSYA and kaempferol. In terms of HSYA content, red safflower exhibited the highest value (2.1736%), followed by yellow safflower (1.5517%) and white safflower (0.0493%), showing a descending order of red > yellow > white. Significant differences in HSYA content were observed among all three cultivars (*p* < 0.05). Regarding kaempferol content, white safflower showed the highest value (3.5781%), followed by yellow (0.2045%) and red (0.1594%) safflower, exhibiting an opposite trend of white > yellow > red. The kaempferol content in white safflower was significantly higher than that in both red and yellow safflower (*p* < 0.05). For kaempferol-3-O-rutinoside content, white safflower contained the highest level (36.2403%), followed by red (9.6354%) and yellow (6.3351%) safflower, showing a distinct distribution pattern of white >> red > yellow. Significant differences in kaempferol-3-O-rutinoside content were detected among all three cultivars (*p* < 0.05).

### 3.2. Results of Network Pharmacology Analysis

#### 3.2.1. Screening of Core Targets Related to α-Glucosidase for the Main Active Components of Safflower

**Figure 2:**
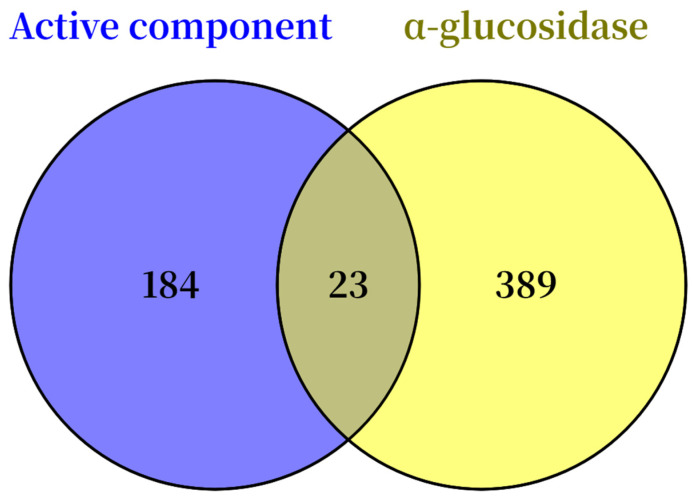
Venn diagram of the main components of safflower and α-glucosidase targets.

A total of 207 targets of the three main active components of safflower were collected from the SwissTargetPrediction database, and 421 α-glucosidase-related targets were collected from the GeneCards database. The intersecting targets between the active components and α-glucosidase were obtained using the Venny 2.1 online tool (Figure 2). A total of 23 overlapping targets were identified between the active component targets and the α-glucosidase targets.

**Figure 3:**
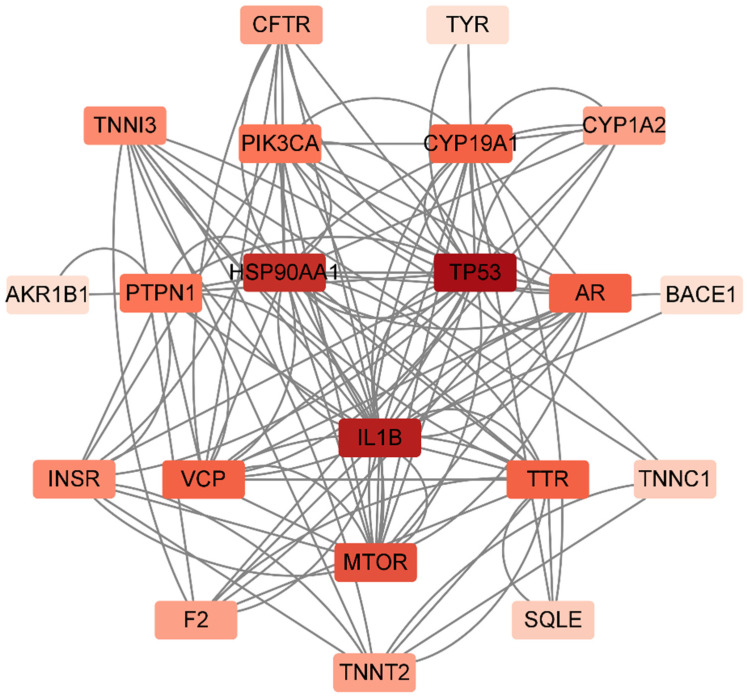
PPI network of intersection targets between major components of safflower and α-glucosidase.

The intersecting targets were uploaded to the STRING online platform to construct a PPI network. The network was then downloaded and imported into Cytoscape software (version 3.9.1) for topological analysis. After removing isolated peripheral targets, 21 targets were obtained. A visualized PPI network diagram was constructed (Figure 3). The degree, betweenness, and closeness values of the intersecting targets are presented in Table 3. The 21 targets were: PIK3CA, VCP, TYR, TNNI3, TNNT2, TNNC1, CFTR, F2, TTR, BACE1, MTOR, TP53, AR, HSP90AA1, CYP19A1, SQLE, INSR, PTPN1, IL1B, AKR1B1, and CYP1A2.

#### 3.2.2. GO Enrichment Analysis

**Figure 4:**
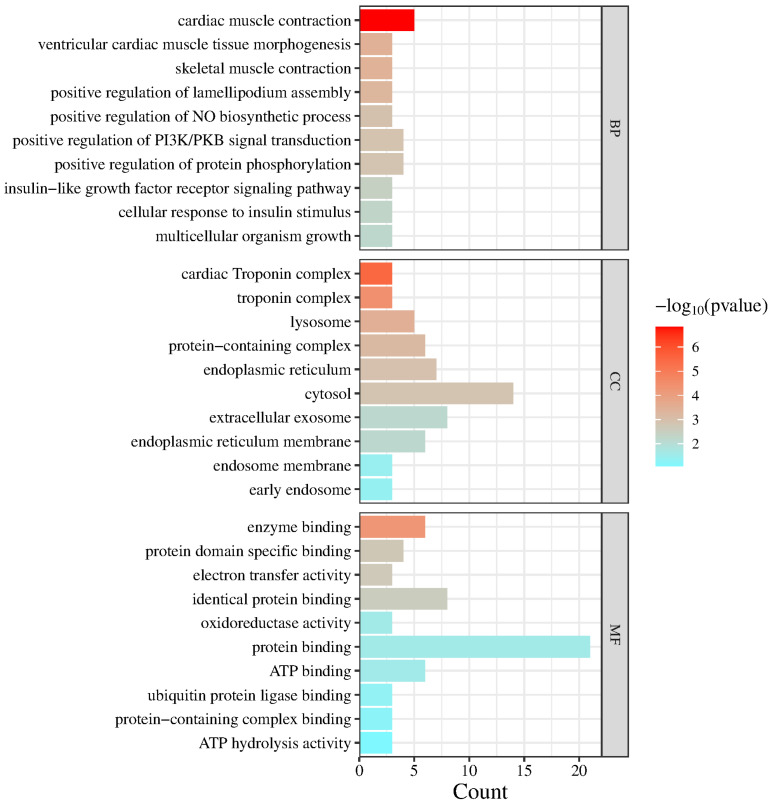
GO enrichment analysis of the main components of safflower and α-glucosidase intersection proteins.

Gene Ontology (GO) enrichment analysis primarily includes biological process (BP), cellular component (CC), and molecular function (MF). GO enrichment analysis of the key targets was performed using the DAVID database, with a significance threshold of *p* < 0.05. A total of 41 enrichment terms were obtained, comprising 18 BP terms, 13 CC terms, and 10 MF terms. The top 10 terms in each category (BP, CC, and MF) ranked by Count value were selected for visualization (Figure 4). The top 10 BP terms were: cardiac muscle contraction, ventricular cardiac muscle tissue morphogenesis, skeletal muscle contraction, positive regulation of lamellipodium assembly, positive regulation of nitric oxide biosynthetic process, positive regulation of phosphatidylinositol 3-kinase/protein kinase B (PI3K/PKB) signal transduction, positive regulation of protein phosphorylation, insulin-like growth factor receptor signaling pathway, cellular response to insulin stimulus, and multicellular organism growth. The top 10 CC terms were: cardiac troponin complex, troponin complex, lysosome, protein-containing complex, endoplasmic reticulum, cytosol, extracellular exosome, endoplasmic reticulum membrane, endosome membrane, and early endosome. The top 10 MF terms were: enzyme binding, protein domain specific binding, electron transfer activity, identical protein binding, oxidoreductase activity, protein binding, ATP binding, ubiquitin protein ligase binding, protein-containing complex binding, and ATP hydrolysis activity.

#### 3.2.3. KEGG Pathway Enrichment Analysis

**Figure 5:**
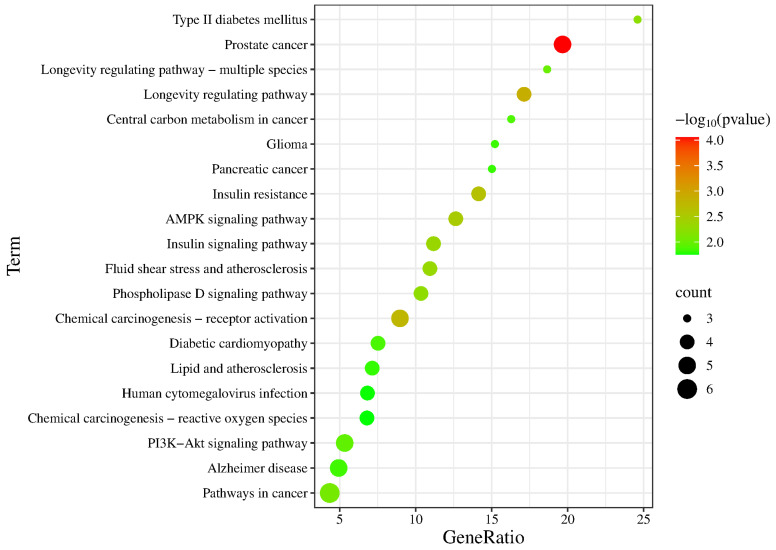
KEGG enrichment analysis of the main components of safflower and α-glucosidase intersection proteins.

KEGG pathway enrichment analysis of the key targets was performed using the DAVID database with a significance threshold of *p* < 0.05, and enriched KEGG pathways were obtained. The top 20 pathways ranked by count value were selected for visualization (Figure 5). In the figure, the *X*-axis represents the percentage of genes, and the *Y*-axis represents the pathway names. The bubble area indicates the number of genes enriched in the pathway (i.e., the larger the bubble, the greater the number of enriched genes). The bubble color represents the magnitude of the *p*-value (the darker the color, the smaller the *p*-value); a smaller *p*-value indicates greater significance of the signaling pathway. The main enriched pathways related to diabetes were type 2 diabetes mellitus, insulin resistance, AMPK signaling pathway, and PI3K-Akt signaling pathway. These pathways suggest that the main components of safflower may have an effect on diabetes.

#### 3.2.4. Active Component–Key Target–Important Pathway Network Diagram

**Figure 6:**
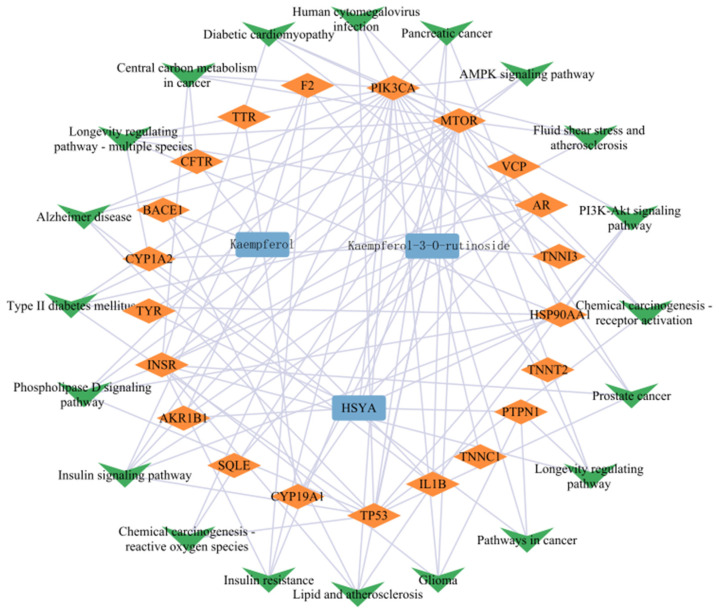
Diagram of the main component–key target–pathway network.

The “active component–key target–important pathway” network diagram of safflower is shown in Figure 6. The network consists of 44 nodes (including 3 main active component nodes, 21 target nodes, and 20 pathway nodes) and 108 edges. The results indicate that the main active components of safflower exhibit multi-target interactions associated with α-glucosidase and act on multiple pathways.

### 3.3. Molecular Docking Results of the Main Active Components of Safflower with α-Glucosidase

Generally, in molecular docking, the lower the binding energy between the macromolecular receptor protein and the small-molecule ligand active component, the more stable the conformation and the greater the likelihood of interaction. A binding energy < 0 kcal/mol indicates that the two molecules can dock under natural conditions, while a binding energy < −5 kcal/mol suggests favorable docking. The binding energies of the positive control acarbose and the three main active components of safflower (HSYA, kaempferol, and kaempferol-3-O-rutinoside) with α-glucosidase are shown in Table 4. As indicated in Table 4, the binding energies of acarbose and the three main active components of safflower with α-glucosidase were all less than −5.0 kcal/mol, demonstrating that they all possess good binding affinity for α-glucosidase, and also validating the reliability of the network pharmacology predictions. The order of binding energies was: kaempferol-3-O-rutinoside < acarbose < HSYA < kaempferol. Notably, kaempferol-3-O-rutinoside exhibited the lowest binding energy with α-glucosidase, even lower than that of acarbose, suggesting that kaempferol-3-O-rutinoside may have strong α-glucosidase inhibitory activity.

**Figure 7:**
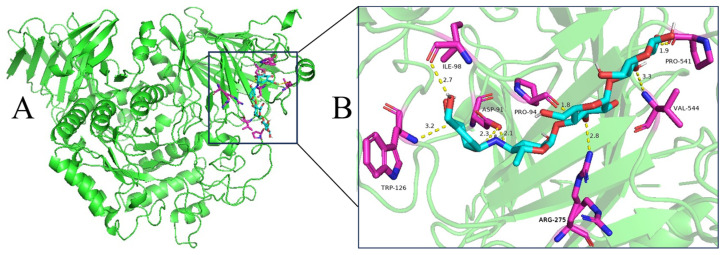
Molecular docking patterns of acarbose and α-glucosidase (**A**) Overall binding conformation of acarbose (green sticks) within the whole enzyme structure; (**B**) Magnified view of the active pocket, showing key residues (magenta sticks, named and numbered), hydrogen bonds (yellow dashed lines), and bond distances (Å).

**Figure 8:**
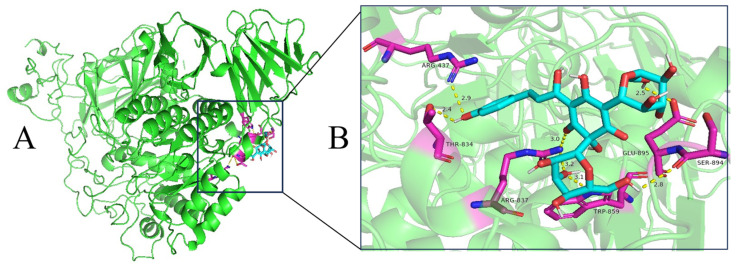
Molecular docking patterns of HSYA and α-glucosidase (**A**) Overall binding conformation of HSYA (green sticks) within the whole enzyme structure; (**B**) Magnified view of the active pocket, showing key residues (magenta sticks, named and numbered), hydrogen bonds (yellow dashed lines), and bond distances (Å).

**Figure 9:**
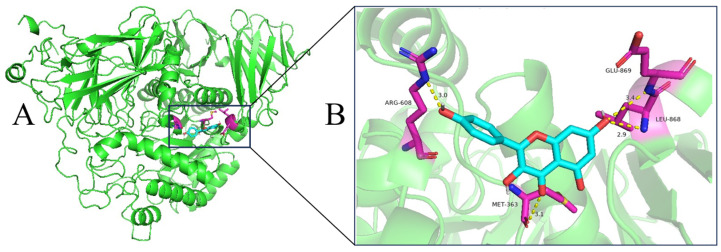
Molecular docking patterns of kaempferol and α-glucosidase (**A**) Overall binding conformation of kaempferol (green sticks) within the whole enzyme structure; (**B**) Magnified view of the active pocket, showing key residues (magenta sticks, named and numbered), hydrogen bonds (yellow dashed lines), and bond distances (Å).

**Figure 10:**
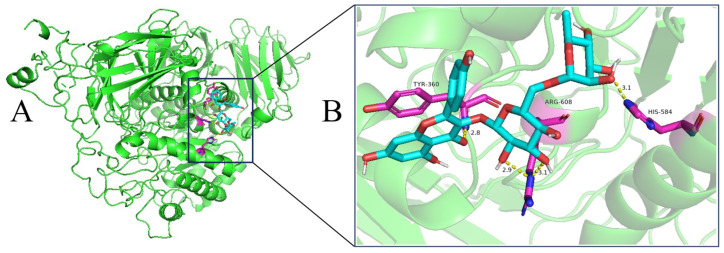
Molecular docking patterns of kaempferol-3-O-rutinoside and α-glucosidase (**A**) Overall binding conformation of kaempferol-3-O-rutinoside (green sticks) within the whole enzyme structure (grey surface); (**B**) Magnified view of the active pocket, showing key residues (magenta sticks, named and numbered), hydrogen bonds (yellow dashed lines), and bond distances (Å).

Using PyMol software, the conformations with the lowest binding energy of acarbose, HSYA, kaempferol, and kaempferol-3-O-rutinoside with α-glucosidase were visualized, as shown in Figure 7, Figure 8, Figure 9 and Figure 10. As shown in Figure 7, acarbose interacts with the amino acid residues TRP-126, ILE-98, ASP-91, PRO-94, PRO-541, VAL-544, and ARG-275 of α-glucosidase. As shown in Figure 8, HSYA interacts with the amino acid residues ARG-437, THR-834, ARG-837, TRP-859, GLU-895, and SER-894 of α-glucosidase. As shown in Figure 9, kaempferol interacts with the amino acid residues ARG-608, MET-363, GLU-869, and LEU-868 of α-glucosidase. As shown in Figure 10, kaempferol-3-O-rutinoside interacts with the amino acid residues TYR-360, ARG-608, and HIS-584 of α-glucosidase.

### 3.4. Results of In Vitro α-Glucosidase Inhibition Assay

**Figure 11:**
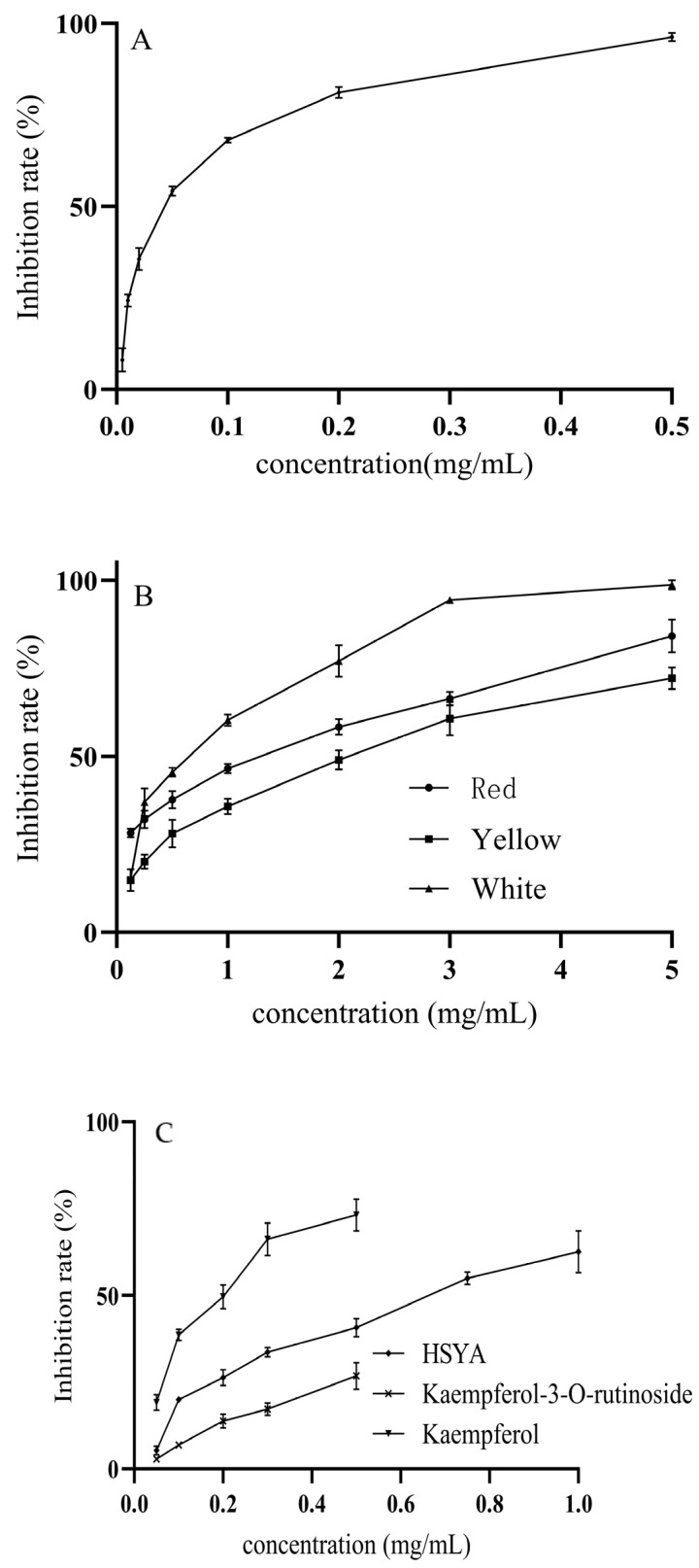
Inhibition of α-glucosidase by acarbose (**A**), safflower extract (**B**) and active ingredients (**C**) (Mean ± SD, *n* = 3) (*p* < 0.05). Note: IC_50_ of kaempferol-3-O-rutinoside could not be determined because the inhibition rate did not exceed 50% at the maximum soluble concentration (0.5 mg/mL).

As shown in Figure 11A, within the concentration range of 0.005–0.5 mg/mL, the inhibitory effect of the positive control acarbose on α-glucosidase increased with increasing concentration, showing a dose-dependent relationship. When the concentration reached 1.0 mg/mL, the increase in inhibition rate diminished. At a concentration of 0.2 mg/mL, the inhibition rate of acarbose against α-glucosidase reached 87.94%, indicating that acarbose exerts a significant inhibitory effect on α-glucosidase. As shown in Figure 11B, the safflower extracts of the three flower colors all exhibited good inhibitory activity against α-glucosidase, and the inhibition rates increased with increasing concentration. In the concentration range of 0.125–5 mg/mL, the relationship between the concentration of the three safflower extracts and the α-glucosidase inhibition rate was dose-dependent. The order of α-glucosidase inhibitory activity among the three flower colors was: white safflower > red safflower > yellow safflower. As shown in Figure 11C, the inhibition rates of HSYA, kaempferol, and kaempferol-3-O-rutinoside against α-glucosidase all increased with increasing concentration. Among them, kaempferol exhibited the highest inhibition rate. In the concentration range of 0.05–0.5 mg/mL, the inhibition rates of kaempferol and kaempferol-3-O-rutinoside showed a dose-dependent relationship with their concentrations. Comparing the three active components, the order of their α-glucosidase inhibitory activity was: kaempferol > HSYA > kaempferol-3-O-rutinoside. Both kaempferol and kaempferol-3-O-rutinoside reached their maximum solubility at 0.5 mg/mL. However, at this concentration, the inhibition rate of kaempferol-3-O-rutinoside did not exceed 50% and remained relatively low, indicating that kaempferol-3-O-rutinoside has weak inhibitory activity against α-glucosidase.

The IC_50_ values of each component were calculated from the inhibition rate curves shown in Figure 11 and are presented in Table 5. As shown in Table 5, among the three active components of safflower, kaempferol exhibited the strongest inhibitory effect on α-glucosidase; among the three flower colors, white safflower showed the strongest inhibitory effect on α-glucosidase. Figure 2 shows that white safflower had the highest content of kaempferol, confirming that kaempferol is the main active component responsible for the α-glucosidase inhibitory activity of safflower. Furthermore, although red safflower had a relatively low content of kaempferol, it possessed a high content of HSYA, whereas yellow safflower had low contents of both kaempferol and HSYA. Therefore, the order of α-glucosidase inhibitory activity among safflower with different flower colors was: white safflower > red safflower > yellow safflower.

## 4. Discussion

Research conducted by YAN et al. [25] indicates that the flower color of safflower is typically orange-red; however, due to natural variation, trait segregation occurs in safflower, manifesting as distinct phenotypes including spiny, slightly spiny, and spineless forms as well as divergent flower colors. Correspondingly, the contents of HSYA, kaempferol, and kaempferol-3-O-rutinoside exhibit certain degrees of variation. The literature reports that flower color undergoes alterations across different flowering stages in diverse safflower cultivars, and that the contents of flavonoid constituents in safflower flowers of different colors also display certain differences [26]. Furthermore, investigations have revealed that the flower color variation in safflower is primarily attributed to differences in the flux of the flavonoid biosynthesis pathway and the accumulation levels of anthocyanins and carotenoids. In white flowers, the metabolic flow is directed upstream toward naringenin and colorless flavonoids (e.g., apigenin and kaempferol). In contrast, red flowers show enhanced downstream flow toward eriodictyol and anthocyanins, with significantly higher anthocyanin and carotenoid contents. Yellow flowers accumulate higher levels of isorhamnetin and other yellow-associated metabolites. These differences are regulated by the differential expression of key structural genes (e.g., CHS, F3H, ANS, BZ1), leading to distinct metabolite profiles and ultimately resulting in red, yellow, and white flower colors [27,28].

The chemical composition of white safflower flowers differs substantially from that of red safflower flowers; in particular, the contents of red pigment components are markedly reduced or completely absent in white safflower flowers, whereas the contents of other flavonoids possessing considerable medicinal value, such as kaempferol and kaempferol-3-O-rutinoside, are both significantly higher than those detected in red and yellow safflower flowers [29]. The results of the present study also indicate that the higher the HSYA content, the lower the kaempferol content, the content of kaempferol-3-O-rutinoside is positively correlated with kaempferol content.

In the present study, molecular docking and in vitro experimental assays yielded divergent outcomes. In the molecular docking results, kaempferol-3-O-rutinoside exhibited the lowest binding energy upon docking with α-glucosidase, and this value was even lower than that obtained for acarbose. However, in the actual in vitro enzyme inhibition assay, kaempferol-3-O-rutinoside demonstrated the weakest inhibitory effect. The primary reason for this discrepancy lies in the fact that molecular docking is a computational prediction based on static protein conformations and predefined force field parameters; therefore, it cannot fully simulate the dynamic alterations of the enzyme within the authentic physiological environment of the organism. Under actual circumstances, intermolecular binding involves complex interactions rather than simple rigid docking. Consequently, the definitive conclusions must be grounded in the results of in vivo and in vitro functional assays, while molecular docking can only serve as a reference [30]. Huang et al. [31] illustrated the critical impact of protein flexibility on binding simulations by comparing the performance of a semiflexible model restrained by a single reference structure with that of an enhanced flexible model restrained by two reference structures when docking the kinase inhibitor balanol.

**Figure 12:**
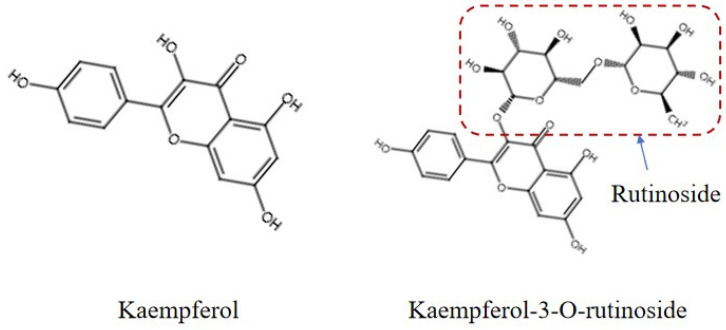
Chemical structures of kaempferol and kaempferol-3-O-rutinoside (the structure within the red box denotes rutinoside).

There exists a close relationship between molecular structure and function. The results of the present study indicate that the inhibitory effects of three flavonoid active constituents from safflower against α-glucosidase follow the rank order: kaempferol > HSYA > kaempferol-3-O-rutinoside. Research conducted by Qin et al. has demonstrated that when the phenolic hydroxyl group at the C-3 position of a flavonoid compound is substituted by a sugar moiety to form a glycosidic bond, the resultant α-glucosidase inhibitory activity is markedly attenuated [32]. As illustrated in Figure 12, kaempferol-3-O-rutinoside is a flavonoid glycoside derived from kaempferol via the substitution of the phenolic hydroxyl group at the C-3 position with a rutinose moiety [13]. Consequently, its inhibitory activity against α-glucosidase is considerably weaker than that of kaempferol, a finding that is entirely consistent with the structure–activity relationships summarized in the literature.

A comparison of the α-glucosidase inhibitory activities of safflower derived from three distinct flower colors revealed that the α-glucosidase inhibitory activities of safflower differ substantially depending on flower color. The results of the present study demonstrate that the inhibitory effects of the three major active constituents in safflower against α-glucosidase follow the rank order: kaempferol (IC_50_ = 0.1768) > HSYA (IC_50_ = 0.6235) > kaempferol-3-O-rutinoside (IC_50_ not determined). Owing to the fact that the kaempferol content in white safflower is substantially higher than that in the other two flower color variants, white safflower exhibits the strongest α-glucosidase inhibitory activity. Although the kaempferol content in red safflower is considerably lower than that in white safflower, its HSYA content is markedly higher than that in white safflower, thereby endowing it with relatively robust α-glucosidase inhibitory capacity. In contrast, the contents of both kaempferol and HSYA in yellow safflower are relatively low, which accounts for its weakest α-glucosidase inhibitory activity. When screening the hypoglycemic activity of safflower active fractions, PENG et al. and XU et al. both found that the active constituents kaempferol and HSYA both exerted certain inhibitory effects on α-glucosidase, and that the inhibitory effect of kaempferol on α-glucosidase was stronger than that of HSYA, which is consistent with the results obtained in the present study [33,34].

## 5. Conclusions

This study revealed significant differences in the content of active ingredients among different colored safflower varieties and their varying α-glucosidase inhibitory activities. White safflower exhibited the strongest α-glucosidase inhibitory activity due to its higher kaempferol content. Kaempferol was identified as the primary active component responsible for α-glucosidase inhibition in safflower, demonstrating stronger effects than HSYA and kaempferol-3-O-rutinoside. The observed discrepancies between molecular docking and in vitro experimental results emphasize that molecular docking predictions should be validated by functional assays. Overall, these findings identify white safflower (Xinhonghua No. 7) and kaempferol as promising candidates for hypoglycemic functional food development, providing a scientific basis for the targeted utilization of Yumin County safflower cultivars. However, the broader anti-diabetic implications suggested by network pharmacology require further validation through in vivo studies, which represent the focus of our ongoing research.

## Figures and Tables

**Table 1:** Linear regression equations for the three control substances.

Compound	Regression Equations	*r*	Linear Range (μg/mL)
HSYA	y = 57617x − 253669	0.9998	4.95~296.7
kaempferol	y = 82149x − 177249	0.9995	6.12~71.40
kaempferol-3-O-rutinoside	y = 589.29x + 3290	0.9996	6.55~131.04

**Table 2:** Results of content determination (Mean ± SD, *n* = 3).

Safflower	Compound Content		
	HSYA(%)	Kaempferol (%)	Kaempferol-3-O-rutinoside (%)
Red (Yuhong No. 1)	2.1736 ± 0.0564 ^a^	0.1594 ± 0.0358 ^b^	9.6354 ± 0.3224 ^b^
Yellow (Xinhonghua No. 4)	1.5517 ± 0.0240 ^a^	0.2045 ± 0.1012 ^b^	6.3351 ± 0.2159 ^c^
White (Xinhonghua No. 7)	0.0493 ± 0.0251 ^b^	3.5781 ± 0.0283 ^a^	36.2403 ± 0.2329 ^a^

Note: Different superscript letters (a, b, c) within the same column indicate significant differences (*p* < 0.05).

**Table 3:** Topological parameters of intersection targets of main components of safflower and α-glucosidase.

Targets	Full Name of the Targets	Degree	Betweenness	Closeness
TP53	TP53: Tumor protein p53	24	0.18277	0.689655
IL1B	Interleukin-1 beta	22	0.26608	0.689655
HSP90AA1	Heat shock protein 90 alpha family class A member 1	20	0.06920	0.645161
MTOR	Mechanistic target of rapamycin	16	0.02280	0.588235
AR	Androgen receptor	14	0.00889	0.555556
VCP	Valosin-containing protein (p97)	14	0.02724	0.571429
CYP19A1	Cytochrome P450 19A1	14	0.05903	0.555556
TTR	Transthyretin	14	0.10325	0.571429
PTPN1	Protein tyrosine phosphatase non-receptor type 1	12	0.10896	0.555556
PIK3CA	Phosphatidylinositol-4,5-bisphosphate 3-kinase catalytic subunit alpha	12	0.00865	0.512821
TNNI3	Troponin I3, cardiac type	10	0.07417	0.526316
INSR	Insulin receptor	10	0.06362	0.526316
CFTR	Cystic fibrosis transmembrane conductance regulator	8	0.00150	0.500000
CYP1A2	Cytochrome P450 1A2	8	0.01316	0.500000
F2	Coagulation factor II (prothrombin)	8	0.01842	0.500000
TNNT2	Troponin T2, cardiac type	8	0.04768	0.465116
SQLE	Squalene epoxidase	4	0.00351	0.434783
TNNC1	Troponin C1, slow skeletal and cardiac type	4	0	0.370370
AKR1B1	Aldo-keto reductase family 1 member B1	2	0	0.363636
BACE1	Beta-secretase 1 (beta-site amyloid precursor protein cleaving enzyme 1)	2	0	0.416667
TYR	Tyrosinase	2	0	0.416667

**Table 4:** Molecular docking results.

Active Ingredients	Acarbose	HSYA	Kaempferol	Kaempferol-3-O-rutinoside
the lowest binding energy (kcal/mol)	−7.8	−7.5	−7.4	−9.3

**Table 5:** IC_50_ of safflower extract and active ingredients for α-glucosidase.

Sample	Acarbose	HSYA	Kaempferol	Kaempferol-3-O-rutinoside	Red Safflower	Yellow Safflower	White Safflower
IC_50_ (mg/mL)	0.0411	0.6235	0.1768	Not determined	0.8695	1.7077	0.5485

## Data Availability

The original contributions presented in this study are included in the Supplementary Material. Further inquiries can be directed to the corresponding author.

## Supplementary Materials

The following supporting information can be downloaded at: https://www.mdpi.com/article/10.3390/cimb48070746/s1.
